# Nature-inspired composite consisting of activated carbon derived from date palm kernel and poly(aniline-*co*-pyrrole) copolymer as electrode for energy storage devices

**DOI:** 10.1038/s41598-026-49903-3

**Published:** 2026-04-27

**Authors:** Shayan Kalavani, Atefeh Kiani, Alireza Alizadeh, Ebrahim Ahmadi

**Affiliations:** Department of Chemistry, Faculty of Science, University of Zanjan, P.O. Box 45195-313, Zanjan, Iran

**Keywords:** Supercapacitor, Composite electrode, Activated carbon, Copolymer, Polyaniline, Polypyrrole, Energy storage, Chemistry, Energy science and technology, Environmental sciences, Materials science

## Abstract

Addressing global environmental challenges and transitioning towards sustainable energy requires the development of advanced and low-cost energy storage materials. This study presents a novel approach for fabricating an electrode composite made from activated carbon based on date palm kernel (ACP) and a polyaniline-*co*-pyrrole (PANI-*co*-Ppy) copolymer using date palm kernel biomass waste. The porous carbon was obtained by carbonization and thermal activation and served as a substrate for the copolymer, synthesized through oxidative polymerization. ACP@PANI-*co*-Ppy composites were prepared at two aniline:pyrrole molar ratios of 1:1 and 1:3 and subsequently characterized through various structural and morphological analyses. Electrochemical performance was assessed by cyclic voltammetry (CV), galvanostatic charge-discharge (GCD), and electrochemical impedance spectroscopy (EIS) in a 3 M KOH electrolyte. ACP@PANI-*co*-Ppy (1:3) composite exhibited the highest specific capacitance of 65.03 F g⁻^1^ at 50 mV s⁻^1^, along with an energy density of 6.7 Wh kg⁻^1^, power density of 312.2 W kg⁻^1^, and outstanding cycling stability with 99.5% retention after 2000 cycles. Additional tests in a two-electrode system using a Na₂SO₄:H₂SO₄ (1 M, 1:1 volume ratio (v/v)) electrolyte showed a specific capacitance of 0.84 F g⁻^1^, energy density of 0.11 Wh kg⁻^1^, and power density of 100 W kg⁻^1^. Although performance was lower compared to the three-electrode setup, the results still confirmed its potential for supercapacitor applications. This study demonstrates an environmentally friendly and cost-effective strategy for transforming agricultural waste into high-performance materials, offering a promising path toward sustainable supercapacitor development. The ACP matrix acts as a mechanical buffer, effectively accommodating the volumetric strain of the copolymer during doping/de-doping processes, which is the key technical novelty of this work.

**Supplementary Information:**

The online version contains supplementary material available at 10.1038/s41598-026-49903-3.

## Introduction

These days, the demand for fossil fuel energy has increased due to population expansion and social economy development. However, the main problems facing human society are pollution, global warming, and the depletion of non-renewable resources^1^. Using electrochemical energy storage devices like supercapacitors (SCs), lithium batteries, and fuel cells which are cost-effective, vastly efficient, and environmentally friendly, can solve the existing challenges^2^.

Low cost, high operational stability, high power density, specific capacitance, extended cycle life, and fast charge/discharge time are some of the characteristics of supercapacitors, which are devices with high energy storage capabilities^3^. These days, they are being utilized more and more in portable electronics, electric cars, and aircraft. SCs fall into two distinct categories: pseudocapacitors and electric double-layer capacitors (EDLC). By quickly absorbing and expelling electrolyte ions from the electrode surface, EDLCs store energy^4^. Meanwhile, a subclass of pseudocapacitors uses faradaic redox processes close to the electrode surface to store charges^2^. Active materials for preparing SC electrodes are carbon materials, conductive polymers, and metal oxides^5^. Carbon materials have excellent conductivity owing to their harmonic attributes, such as high porosity, great surface area, distribution, pore size, and surface reactivity. Environmental problems are raised by the fact that fossil fuels now make up the majority of activated carbon (AC) precursors. Renewable biomass that is acquired from agricultural waste has been considered a prolific carbon source that can be employed in SCs for energy storage and conversion^6,7^. However, the poor energy density, low capacitance and surface energy storage of EDLC materials are some of the main problems of ACs^8^. To untangle this problem, electrode materials with quasi-capacitive energy storage properties have been used^9^. Among different types of conductive conjugated polymers such as poly(3-methylthiophene) )PMTh(, polythiophene )PTh(, polyaniline )PANI(, polypyrrole )Ppy(, and poly(3,4-ethyleneoxythiophene) )PEDOT(^10^, PANI and Ppy are highly investigated as appropriate candidates with inimitable characteristics such as a high pseudocapacitance, ease of synthesis, low cost, controllable morphology, and good conductivity. Now, *in situ* chemical oxidative polymerization is one of the conventional techniques to produce poly(aniline-*co*-pyrrole)^11^. The mechanism of energy storage in PANI takes place through redox reactions^12,13^. Copolymers based on polyaniline and polypyrrole are employed to prepare composite materials appropriate for electrodes with promising applications in supercapacitors due to their suitable surface electrical conductivity, compatibility with the internal stress of electrodes, and electrical contact between conductive agents and particles^9,14,15^. Vikas Kumar Pandey et al. oxidative polymerization to create a PANI/AC/CuF composite. The composite demonstrated an energy density of 49.6 Wh kg^-1^ and a specific capacity of 248.3 F g^-1^ at 1 A g^-1^^16^. A carbon nanotube woven film (CNWF)/PANI composite was created by Bingjian Li et al. using cyclic voltammetry electrochemical polymerization. It has a high specific capacity of 1098.0 F g^-1^ at 1 A g^-1^ and an energy density of 54.9 Wh kg^-1^^14^. A Ppy@NPCNFs (N-doped porous carbon nanofiber skeleton) composite bearing an energy density of 3.10 Wh kg^-1^ and a specific capacity of 342.1 F g^-1^ at 1 A g^-1^ was developed by Lulu Gao et al.^17^. In this study, the ACP@PANI-*co*-Ppy (1:3) composite was fabricated as a pseudocapacitor electrode *via* carbonization and activation followed by oxidative polymerization, which has not been reported yet. For the first time, active carbon derived from date palm kernel (ACP) was made by calcining date palm kernel biomass waste at high temperature after its activating with ZnCl_2_. Then, ACP was coated with poly(aniline-*co*-pyrrole) (PANI-*co*-Ppy) copolymer at two aniline:pyrrole molar ratios of 1:1 and 1:3 to prepare novel electrode materials. ACP@PANI-*co*-Ppy (1:3) composite showed high conductivity, unique electrochemical performance, and appropriate energy density. This composite can play an important role in forming a new generation of quasi-capacitive electrolytic capacitors because it is efficient, stable, and cost-effective.

## Experimental

### Materials

ZnCl_2_ (98%), KOH (98%), NaOH (98%), FeCl_3_ (98%), ammonium persulfate (APS), aniline monomer, HCl (37%), HNO_3_ (98%), and ethanol (99%) were purchased from Merck Company, Germany. date palm kernel powder was purchased from the market in West Azarbaijan province, Khoy city. Distilled water was used in all steps. Nickel foam (NF) and polytetrafluoroethylene (PTFE) were purchased from Medical Chemical Foam Company and Redox Kala Company, respectively.

### Characterization

The structural composition of the synthesized compounds was investigated using Fourier transform infrared spectroscopy (FT-IR, Nicolet is10, USA), and their crystallinity was examined using an X-ray diffraction (XRD, Bruker D8) instrument. Field emission scanning electron microscopy (FE-SEM, KYKY EM800F) and transmission electron microscopy (TEM, Philips em208s, 100kv) were used to examine morphological features. The surface chemistry of the materials and the content of elements present in the samples were investigated using FE-SEM, EDS, and Map MIRA3-XMU. We employed an advanced Origaflex potentiostat/galvanostat model to conduct precise and reliable electrochemical measurements.

### Synthesis of ACP

#### Chemical activation by ZnCl_2_ salt

An aqueous solution of zinc chloride in distilled water (30 mL, 1 M) was prepared. The prepared solution was stirred continuously at 70 °C for 30 minutes with date palm kernel powder (2 g). The mixture was rinsed 3 times with distilled water and ethanol by centrifuge and thoroughly dehydrated in a vacuum chamber for 12 hours at 60 °C^18^.

#### Carbonization

Carbonization was the production of carbon from wood by it being burned in the microwave where it is mixed with nitrogen gas. The powder from the preceding step (2 g) was placed in the center of the furnace in a crucible and the temperature was regulated at 500 °C for 3 hours with the rate of 5 °C/min. Then, it was chilled to room temperature. The prepared powder was first refluxed by an aqueous solution (25 mL, 3 M) of NaOH and then HNO_3_ (25 mL, 3 M) at a temperature of 110 ℃ and was subjected to ongoing stirring at a rate of 500 rpm for 6 hours, as well as the sediment was filtered at each step. It was filtered with water and ethanol and then desiccated in a vacuum oven for 12 hours at 60 °C. ACP was a name given to activated carbon black^18^.

### Synthesis of ACP@PANI-*co*-Ppy

The prepared ACP (0.5 g) was added to a solution of aniline (0.77 mL) and pyrrole (2.31 mL) in hydrochloride (50 mL, 1 M) after dispersal through sonication for 15 minutes, and then held in an ice-water bath at 0 °C. Then an aqueous APS solution (10 mL, 1.1 M) was added dropwise as an initiator. FeCl_3_ (2.5 g) was completely dissolved in 50 mL of distilled water, and then dropwise addition of the catalyst solution (25 mL) was given to the mixture to speed up the polymerization. The prepared solution was stirred for 6 hours. Then, the sediment was washed with ethanol as well as distilled water, and it was desiccated in a vacuum oven for 12 hours at 70 °C. Created precipitate was named ACP@PANI-*co*-Ppy (1:1). Composite ACP@PANI-*co*-Ppy (1:3) with higher content of pyrrole was also prepared at a volume ratio of aniline:pyrrole (1:3).

### Electrode fabrication and electrochemical measurements

Before electrochemical analyses, nickel foam electrodes (1.0 × 1.0 cm) were cut into precise rectangular sections. They were then cleaned in 1.0 M hydrochloric acid and acetone for 15 min and dried for future use. Ethanol was used for complete mixing of the synthesized active materials (80 wt%), activated carbon (10 wt%), and polytetrafluoroethylene (10 wt%). After that, the mixture was applied to 1.0 × 1.0 cm nickel foam, desiccated for 12 h at 60 °C, and subjected to a pressure of 10 MPa. This was used as the working electrode^19^. The active mass of the electrode is 0.4 mg cm^-2^. GCD (galvanostatic charge-discharge), EIS (electrochemical impedance spectroscopy), and CV (cyclic voltammetry) measurements were performed using Origa master software. The tests employed an electrolyte of aqueous KOH solution (3 M) and a three-electrode system including a working electrode, a platinum counter, and an Ag/AgCl reference electrode.

## Results and discussion

### Synthesis and characterization of ACP@PANI-*co*-Ppy (1:3)

**Fig. 1:**
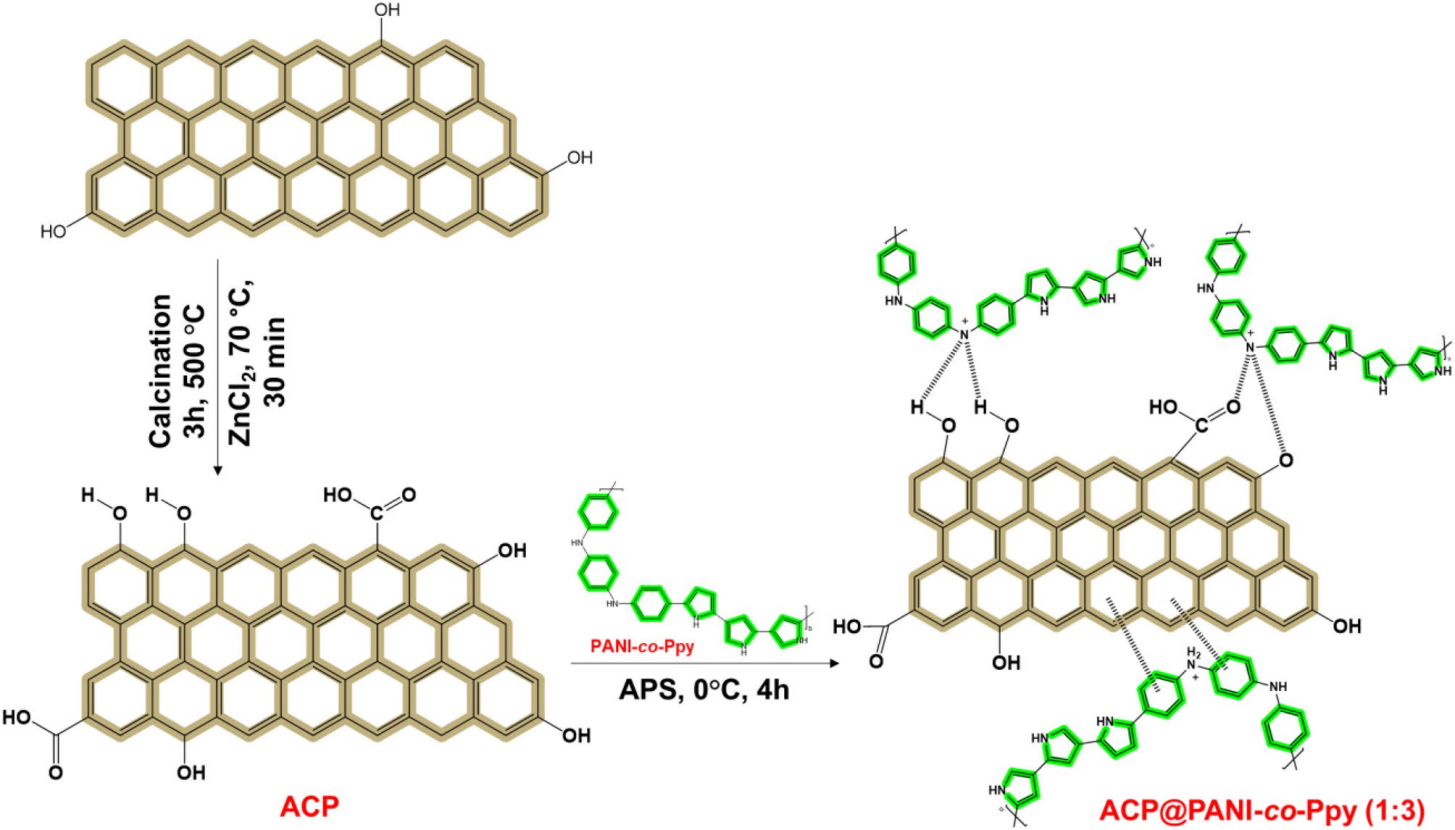
Preparation and molecular structure of ACP@PANI-*co*-Ppy (1:3) composite.

As shown in Fig. 1, After first activating date palm kernel powder with ZnCl_2_ and calcining it at 500 °C in a neutral set-up, ACP was made. Pyrrole is more readily oxidized than aniline due to its higher π-electron delocalization and electron-rich aromatic ring, which facilitates the oxidation process. Unlike, the oxidation of aniline is more difficult as the benzene ring draws the electrons toward itself. So, due to the different electrochemical properties of these monomers, modifying their molar ratio and controlling the reaction during synthesis of PANI-*co*-Ppy copolymer is very important to obtain suitable results. Poly(aniline-*co*-pyrrole) with high conductivity and suitable porosity was uniformly coated on the ACP substrate via a chemical oxidation polymerization process (Fig. S1). The prepared copolymer, due to its high conductivity, provided suitable conditions for ion and electron transport.^20,21^.

**Fig. 2:**
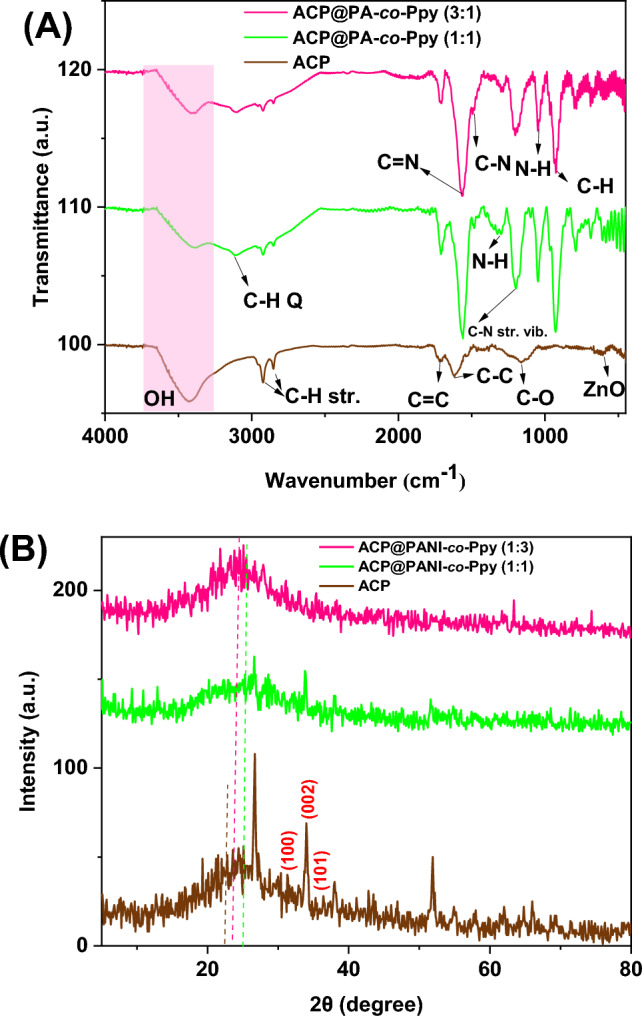
(**A**) FT-IR spectra and (**B**) XRD patterns of the prepared compounds.

The chemical structures of ACP, ACP@PANI-*co*-Ppy (1:1), and ACP@PANI-*co*-Ppy (1:3) were verified by FT-IR spectroscopy (Fig. 2A). All spectra show hydroxyl O-H stretching vibrations in a wide range between 3300 and 3650 cm^-1^. The peaks at 2920 and 2857 cm^-1^ are associated with C-H stretching vibrations. The peak at 1700 cm^-1^ corresponds to the C=C stretching vibrations. Due to the presence of the lignin with aromatic structure in the biomass, the peak at 1620 cm^-1^ relates to the C-C stretching. C-O stretching vibrations in carboxylic acid, ether, alcohol, and ester structures have a peak at 1120 cm^-1^, and the absorption band at the center of 529 cm^-1^ is characterized as a Zn-O bond vibration^22^.

In the spectra of ACP@PANI-*co*-Ppy (1:1) and ACP@PANI-*co*-Ppy (1:3) composites, the characteristic peak at 1568 cm^-1^ is related to the angular transformation of the N-H groups in polypyrrole. The C-N stretching vibration peak at 1490 cm^-1^, the N-H deformation peak at 1045 cm^-1^, and the C-H deformation peak at 927 cm^-1^ appeared. The peaks at 1303 and 1490 cm^-1^ are respectively due to the deformation of the N-H groups and the C-C bond of the benzoid structure, which can be attributed to the doped polyaniline structure in the composites^23^. It also shows a broad peak at 3100 cm^-1^ due to the C-H quinoid stretching vibrations^24^. The emergence of a peak at 1225 cm^-1^ is assigned to the C-N⁺ stretching vibration in the polaron structure, providing definitive proof of successful protonated doping within the copolymer matrix. The presence of peaks corresponding to ACP and the poly(aniline-*co*-pyrrole) copolymer in the FTIR spectra of the final composites, along with changes in peak intensity and shape, indicate the formation of ACP@PANI-*co*-Ppy composites. Fig. S2 shows the FT-IR spectra of pure PANI, Ppy, and their copolymer for better comparison.

XRD analysis of the synthesized materials was recorded in the 2θ range of 10-80° (Fig. 2B). This analysis was used to investigate the crystal structure of the prepared composites. As shown in the XRD pattern of ACP compound, characteristic diffraction peaks of ZnO with 2θ values ​​of 31.80, 34, and 36.4°, respectively correspond to Miller’s plans (100), (002) and (101) suggest a logical synthesis of zinc oxide. Considering that the chemical activation process of the ACP sample was carried out using ZnCl₂, the presence of diffraction peaks related to ZnO can be seen in the XRD pattern; this indicates the formation of a crystalline phase of zinc oxide in the structure of the synthesized material and also the appropriate reactivity of the activating agent during the chemical activation process. Also, ZnO sublimes at 1800 °C and the chemical activation temperature of the sample is 600 °C, as a result, ZnO does not leave the sample and is visible in the XRD spectrum. Also, the highest peak at 2θ ≈ 23° demonstrates that the lignocellulosic form of the biomass has been removed and replaced by carbon^22^. In the XRD pattern of ACP@PANI-*co*-Ppy (1:3), a broad peak at 2θ of 24° displays the amorphous nature of Ppy, which is attributed to the presence of repeating pyrrole units. A broad peak at 2θ of 25° shows the semi-crystalline nature of PANI due to its perpendicular and parallel alternation^24^. The observation of characteristic peaks of the copolymer covered on the ACP surface and the disappearance of ZnO peaks in the XRD diagrams confirm the proper synthesis of these composites. Crystallinity percent was calculated using Equation (1) ref^25^.

1 $$\mathbf{C}\mathbf{r}\mathbf{y}\mathbf{s}\mathbf{t}\mathbf{a}\mathbf{l}\mathbf{l}\mathbf{i}\mathbf{n}\mathbf{i}\mathbf{t}\mathbf{y}\,\mathbf{p}\mathbf{e}\mathbf{r}\mathbf{c}\mathbf{e}\mathbf{n}\mathbf{t}=\frac{\mathbf{A}\mathbf{r}\mathbf{e}\mathbf{a}\,\mathbf{u}\mathbf{n}\mathbf{d}\mathbf{e}\mathbf{r}\,\mathbf{t}\mathbf{h}\mathbf{e}\,\mathbf{c}\mathbf{r}\mathbf{y}\mathbf{s}\mathbf{t}\mathbf{a}\mathbf{l}\mathbf{l}\mathbf{i}\mathbf{n}\mathbf{e}\,\mathbf{p}\mathbf{e}\mathbf{a}\mathbf{k}\mathbf{s}}{\mathbf{A}\mathbf{r}\mathbf{e}\mathbf{a}\,\mathbf{u}\mathbf{n}\mathbf{d}\mathbf{e}\mathbf{r}\,\mathbf{a}\mathbf{l}\mathbf{l}\,\mathbf{p}\mathbf{e}\mathbf{a}\mathbf{k}\mathbf{s}(\mathbf{c}\mathbf{r}\mathbf{y}\mathbf{s}\mathbf{t}\mathbf{a}\mathbf{l}\mathbf{l}\mathbf{i}\mathbf{n}\mathbf{e}+\mathbf{a}\mathbf{m}\mathbf{o}\mathbf{r}\mathbf{p}\mathbf{h}\mathbf{o}\mathbf{u}\mathbf{s})}\times 100$$

ACP, ACP@PANI-*co*-Ppy (1:1), and ACP@PANI-*co*-Ppy (1:3) showed relative crystallinity of 52.5, 79, and 82.3%, respectively. In the ACP sample, the crystallinity decreased due to the large number of pores in the activated carbon. After coating with PANI-*co*-Ppy, the crystallinity increased due to the abundance of regular copolymer chains on the surface of the activated carbon ^25^. The results confirm that the copolymer chains successfully coated the ACP surface.

**Fig. 3:**
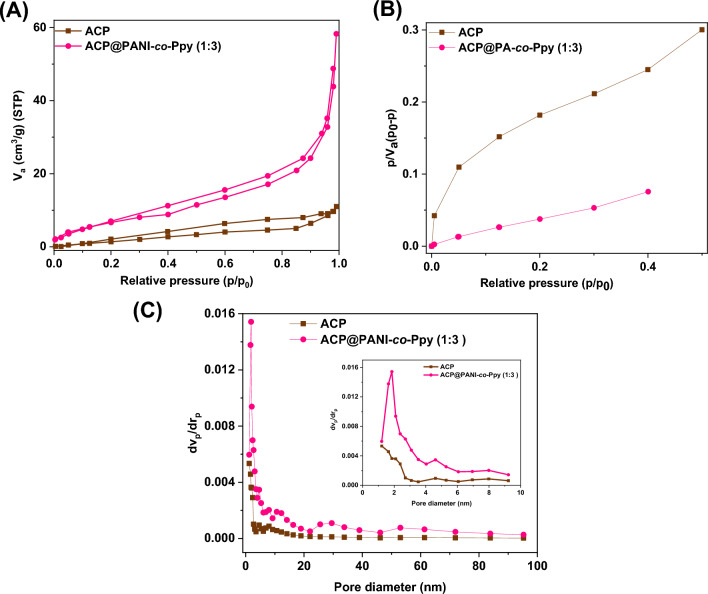
(**A**) Nitrogen adsorption-desorption isotherms; (**B**) BET plots, and (**C**) BJH plots of ACP and ACP@PANI-*co*-Ppy (1:3).

Nitrogen (N₂) adsorption-desorption isotherm analysis was used to investigate the surface area, pore size distribution, and porosity of the synthesized materials (Fig. 3). The isotherm type of IV with H4 hysteresis loop for the ACP, which represents the behavior of mesoporous substances, is shown in Fig. 3A. ACP has surface area of 971 m^2^ g^-1^, which is sufficient for modification using conductive copolymer. The carbon and ZnCl_2_ mixture are heated (calcined) at high temperatures, always above 400 °C. The ZnCl_2_ reacts as an activating agent that increases porosity in the carbon material during the heating. The ZnCl_2_ also helps break down the carbon structure and form further micropores, thus increasing the surface area of the carbon. More surface area is important for the storage of charge in supercapacitors^22^. Decreasing the specific surface area in ACP@PANI-co-Ppy (1:3) to 27.67 m^2^ g^-1^ compared to ACP successfully shows the integration of polymer chains into the porous structure of ACP. The pore volume increased from 0.016 cm^3^ g⁻^1^ for ACP to 0.088 cm^3^ g⁻^1^ for ACP@PANI-*co*-Ppy (1:3), which may be attributed to the formation of a porous structure induced by the coating of the conductive polymer network. The mean pore diameter increased from 6.91 nm for ACP to 12.82 nm for ACP@PANI-*co*-Ppy (1:3) (Figs. 3B and 3C). This 97% reduction is attributed to the "pore-filling effect," where copolymer chains grow within the internal micropores, converting the physical surface into a high-density Faradaic reservoir. Despite the area drop, the mean pore diameter increased from 6.91 nm to 12.82 nm.

**Fig. 4:**
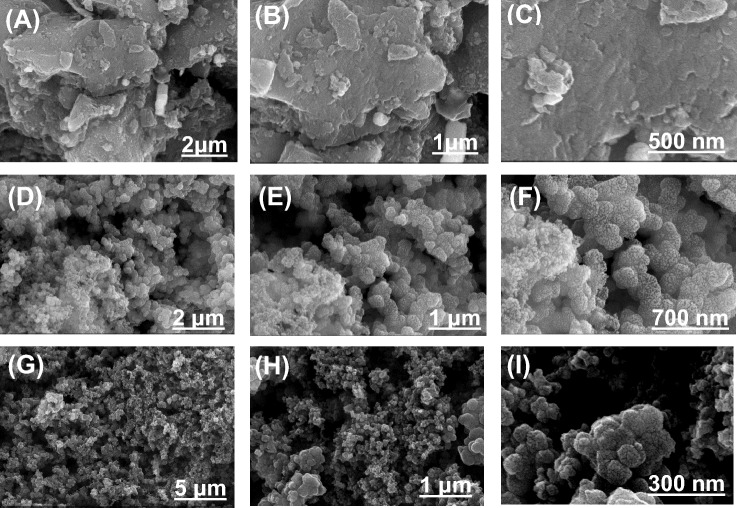
FE-SEM images of (**A**-**C**) ACP at different magnifications, (**D**-**F**) ACP@PANI-*co*-Ppy (1:1) at different magnifications, and (**G**-**I**) ACP@PANI-*co*-Ppy (1:3) at different magnifications.

FE-SEM images (Fig. 4) show the surface morphology of the synthesized materials including ACP, ACP@poly(aniline-*co*-pyrrole) (1:1), and ACP@poly(aniline-*co*-pyrrole) (1:3). It can be seen in the ACP images (Fig. 4A-C) that the surface has roughness mainly due to the removal of water and organic materials in the calcination process. This leads to an increase in surface area, which becomes new sites for electrochemical activity. Also, large wrinkles on the surface can increase both the porosity of the electrode and its electrochemical activity. Also, ACP is composed of rough and uneven surfaces with open and accessible spaces between them. Having these holes in the material enables ions to reach more deeply into the electrode and to interact more easily with the liquid solution which helps the movement of electrical charge throughout the system^7,26^. For ACP@poly(aniline-*co*-pyrrole) composites (Figs. 4D–I), surface accumulation of PANI-*co*-Ppy on the surface of ACP is relatively amorphous and the chain structures are well connected. A larger surface area, many pores and suitable ionic factors can directly boost the energy storage capacity of electrodes. Therefore, because of their appropriate structure, good electrical conductivity, and great ionic accessibility, ACP@poly(aniline-*co*-pyrrole) can work efficiently as materials for supercapacitor electrodes and similar devices.

**Fig. 5:**
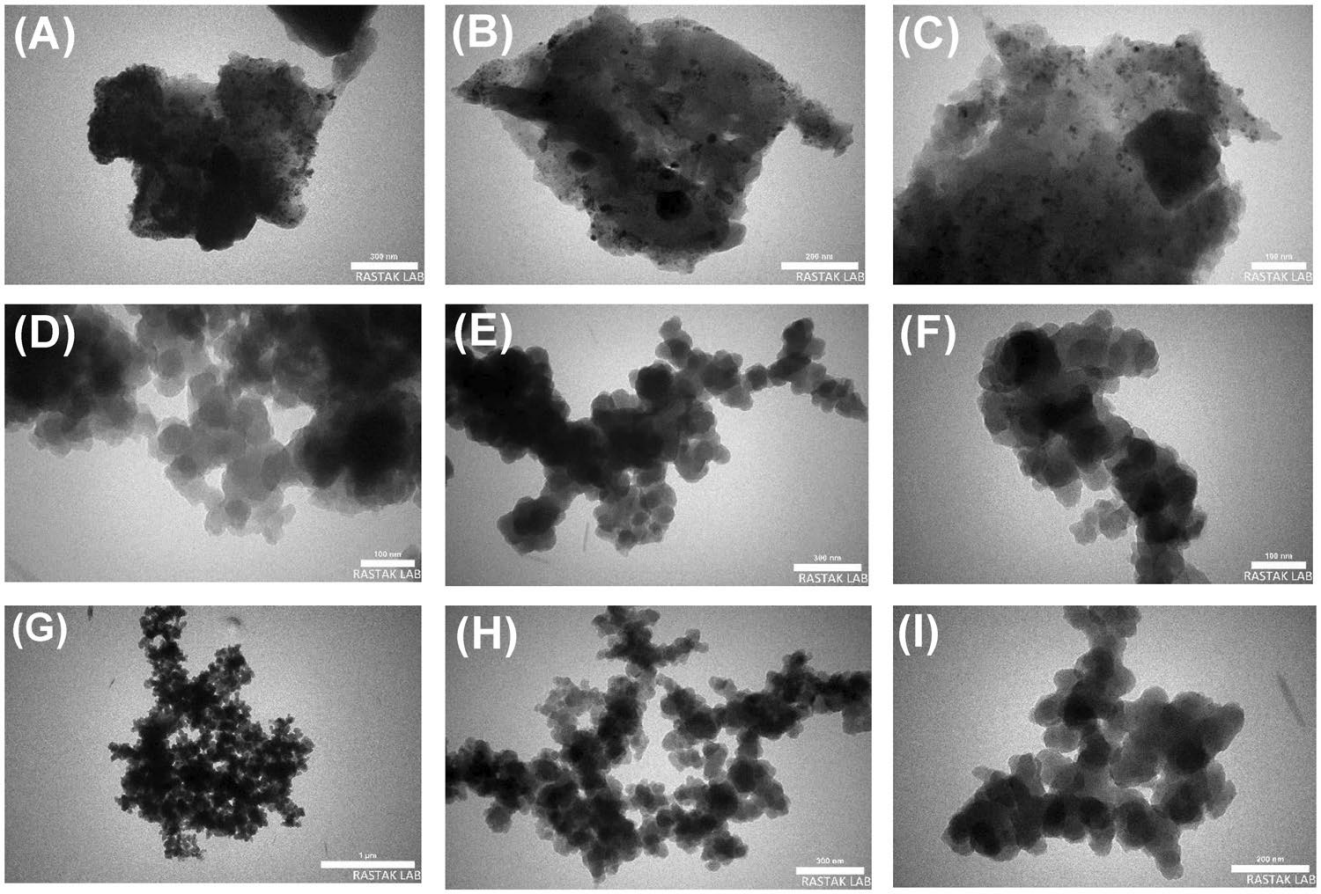
Images of TEM from (**A**-**C**) ACP, (**D**-**F**) ACP@PANI-*co*-Ppy (1:1), and (**G**-**I**) ACP@PANI-*co*-Ppy (1:3).

TEM was also used to determine the morphological characteristics. In Figs. 5A–C, due to the irregular assembly of the carbon network and the presence of amorphous regions in the structure of the ACP sample, dark spots are visible in the (TEM) images, which indicate the lack of disorder in some areas of the material and are also consistent with the images obtained from FE-SEM. The complex three-dimensional porous structure greatly increases the specific surface area and therefore provides a high density of ion adsorption sites and interconnected ion diffusion pathways^27^. In Figs. 5D–I, our claim about the formation of uniform poly(aniline-*co*-pyrrole) on the ACP surface and the successful formation of the composite was reconfirmed. In addition, ACP@PANI-*co*-Ppy (1:1) and ACP@PANI-*co*-Ppy (1:3) composites have a very dense and compact structure. Such a configuration will likely enhance the kinetics of charge storage and ion diffusion significantly^28^.

**Fig. 6:**
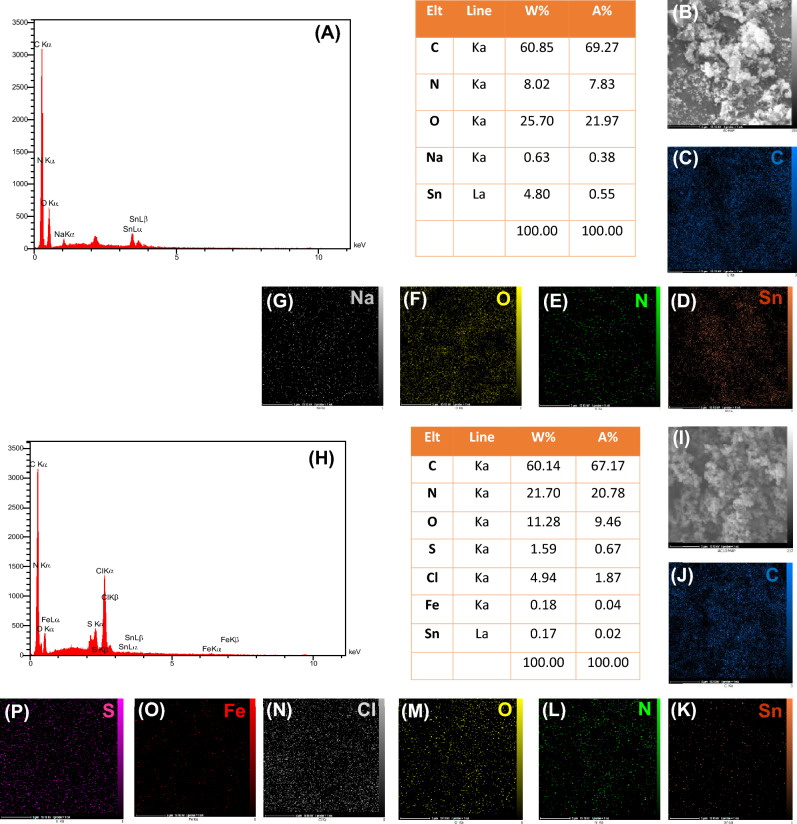
EDS diagram of (**A**) ACP and (H) ACP@PANI-*co*-Ppy (1:3). Elemental mapping of (**B**-**G**) ACP and (**I**-**P**) ACP@PANI-*co*-Ppy (1:3).

Energy dispersive spectroscopy (EDS) and elemental mapping studies were performed to provide detailed observations of the produced ACP and ACP@PANI-*co*-Ppy (1:3) compounds. The calcined composition of ACP has elements of tin (Sn), carbon (C), oxygen (O), sodium (Na), and nitrogen (N) according to the results of EDS analysis (Figs. 6A, 6H). Elemental mapping also shows the presence of oxygen (O), chlorine (Cl), nitrogen (N), sulfur (S), carbon (C), iron (Fe) as well as tin (Sn) in the ACP (Fig. 6B-G) and ACP@PANI-co-Ppy (1:3) composite (Fig. 6I-P). It can be concluded from decreasing the oxygen content and increasing the nitrogen content, that the copolymers cover the surface of ACP homogeneously. Analysis of elemental mapping also confirms this result. In the carbonization process of date palm kernel at 500°C, the decomposition of organic compounds is not complete and some oxygen-containing functional groups such as –COOH, –OH and C=O remain.

The presence of heat-resistant lignocelluloses, residual moisture or contact with air also increases the oxygen content. For this reason, a high oxygen percentage (25.7%) was observed in the ACP sample^29^.

### Electrochemical studies

#### Electrochemical performance of ACP@PANI-*co*-Ppy (1:3) composite

**Fig. 7:**
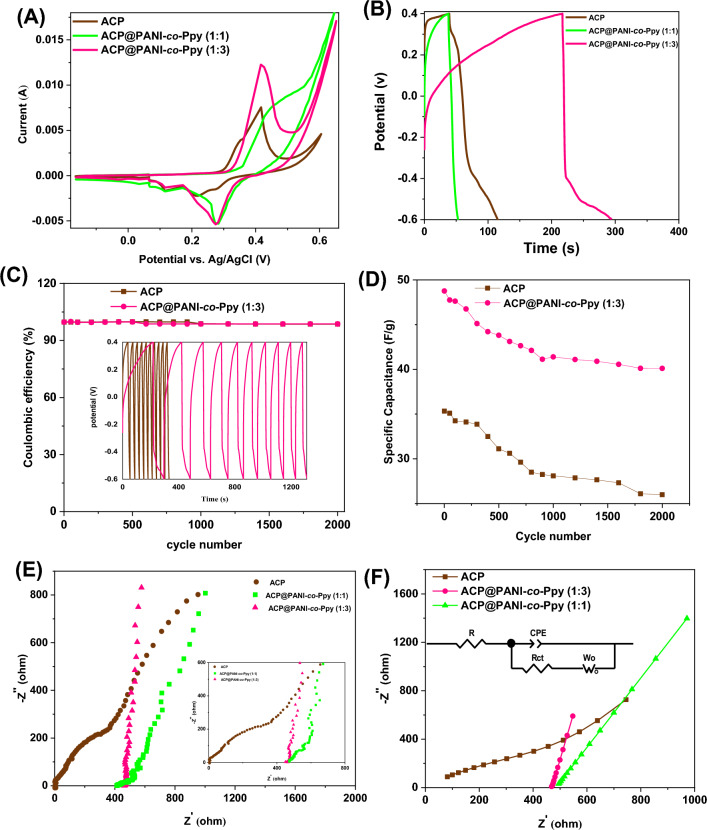
Electrochemical tests were performed in a 3-electrode system with KOH electrolyte (3.0 M). (**A**) CV curves of all compounds prepared at a scan rate of 50 Mv s^-1^: (**B**) GCD curves of all compounds at 0.6 A g^-1^. (**C**) Coulombic efficiency versus cycle number of ACP and ACP@PANI-*co*-Ppy (1:3) electrodes. (**D**) Spinning performance of ACP electrodes as well as ACP@PA-*co*-Ppy (1:3) at a current density of 0.6 A g^-1^. (E) Nyquist plots of all prepared compounds at 1-100 kHz. The inset in (E) shows the magnified high-frequency region (F) shows the R-value of the solution resistivity in the figure for the ACP@PANI-*co*-Ppy (1:3) composite.

ACP@PANI-*co*-Ppy (1:3) composite was tested as a pseudocapacitor electrode material using a three-electrode cell in the electrolyte KOH 3.0 M. CV, EIS, and GCD plots were measured. The CV curve of ACP@PANI-*co*-Ppy (1:3) composite is shown in Fig. 7A. For better comparison, CV measurements were also performed for ACP and ACP@PANI-*co*-Ppy (1:1). All three CV curves show two pairs of distinct redox peaks, which indicate the redox reactions at the electrode surface and the quasi-capacitive properties during the electrochemical process. Redox reactions are shown in Fig. S3. In addition, the level of the integrated CV plot in the ACP@PANI-*co*-Ppy (1:3) electrode is more remarkable than that of the ACP and ACP@PANI-*co*-Ppy (1:1) electrodes. This shows that the ACP@PANI-*co*-Ppy (1:3) composite has more electrochemical activity and a higher capacitance. Specific capacitance assessed by CV using Equation (2):

2 $${\mathrm{C}}_{{\mathrm{x}}} = \frac{{\mathrm{A}}}{{2{\mathrm{mK}}\left( {{\Delta V}} \right)}}$$

In Equation (2), A, m, ΔV, K, and C_x_ are the internal surface of the CV curves, the weight of active material on electrode (g), the value of potential window (V), scanning rate (mV s^-1^), and specific capacitance value (F g^-1^), respectively^30^. From analysis of CV diagrams, it is estimated that ACP, ACP@PANI-*co*-Ppy (1:1), and ACP@PANI-*co*-Ppy (1:3) samples respectively exhibit specific capacitance values ​​of 42.5, 59.1, and 65.03 F g^-1^ at scanning rate of 50 mV.s^-1^. These results clearly show the difference in specific capacitance value among different materials^31^.

Galvanostatic charge/discharge (GCD) curves are commonly exploit to determine specific capacitance of materials and this method is widely known and accepted. Equation (3) is used to calculate value of the specific capacity of several electrodes (F g^-1^) under analysis of galvanostatic charge/discharge curves^32^.

3 $${\mathrm{C}} = \frac{{{\mathrm{I}} \times {\Delta t}}}{{{\mathrm{m}} \times {\Delta V}}}$$

Where Δt, m, I, and ΔV are discharge time (s), mass of active material used in the working electrode (g), discharge current (A), discharge time (s) as well as potential window (V), respectively. The GCD technique shows that the electrode materials mainly rely on Faradaic redox reactions for energy storage^31^. The charge/discharge curves show asymmetry, which is related to the Faradaic capacity of poly(aniline-*co*-pyrrole)^33^. Comparison of three electrodes at a current density of 0.6 A g^-1^ is presented in Fig. 7B. ACP@PANI-*co*-Ppy (1:3) electrode shows the longest discharge time as well as a highest specific capacitance of 48.335 F g^-1^, which is higher than the values ​​of 8.7 F g^-1^ for ACP and 47.2 F g^-1^ for ACP@poly(aniline-*co*-pyrrole) (1:1). Equation (4) has been used to calculate the Coulomb efficiency^34^. According to Fig. 7C, at a current density of 0.6 A g^-1^, ACP and ACP@PANI-*co*-Ppy (1:1) electrodes exhibited the Coulombic efficiency of 99 and 98%, respectively^35^.

4 $$\upeta =\frac{{\mathrm{t}}_{\mathrm{d}}}{{\mathrm{t}}_{\mathrm{c}}}\times 100$$

η, t_d_, and t_c_ are the coulombic efficiency, discharge time and charge time (s), respectively ^34^. It was observed that ACP, ACP@PANI-*co*-Ppy (1:1) and ACP@PANI-*co*-Ppy (1:3) electrodes maintained specific capacitance equal to 75%, 97.3% and 99% after 2000 cycles. The value of specific capacitance decreased in the first 1000 cycles but then approximately stabilizes (Fig. 7D). One of the agents that improve the cyclic performance of composites is the tendance of N containing functional groups, which are present in a large amount in the synthesized copolymer and leads to the easy passage of electrons on the polymer chain. In addition, it is also one of the factors that intensify the pseudo-capacitance effects.

While some sources suggest that KOH could lead to long-term degradation in certain conductive polymers, our experimental findings demonstrate exceptional and sustained performance of the prepared copolymer blend in a 3.0 M alkaline KOH electrolyte. For further investigation, GCD, CV and EIS tests were performed in the three-electrode system and showed that the ACP@PANI-*co*-Ppy (1:3) electrode has the longest discharge time and the highest specific capacitance (48.34 F g⁻^1^); which is clearly higher than the ACP samples (8.7 F g⁻^1^) and the ACP@PANI-*co*-Ppy (1:1) composite (47.2 F g⁻^1^). These results indicated that the higher ratio of polypyrrole in the copolymer structure has contributed to more efficient charge transfer and increased pseudocapacitor capacity.

To further confirm the stability and performance adaptability to non-alkaline environments, experiments were also conducted in a two-electrode system with a mixed electrolyte of Na₂SO₄:H₂SO₄ (1 M, 1:1 volume ratio (v/v)). The ACP@PANI-*co*-Ppy (1:3) electrode once again showed the best performance, achieving a specific capacitance of 0.84 F g⁻^1^, an energy density of 0.117 Wh kg⁻^1^, and a power density of 100 W kg⁻^1^, outperforming the other samples. In comparison, the pure activated carbon (ACP) only delivered a capacitance of 0.26 F g⁻^1^, an energy density of 0.036 Wh kg⁻^1^, and a power density of 99.69 W kg⁻^1^. Also, the ACP@PANI-*co*-Ppy (1:1) composite showed a significant performance drop (0.116 F g⁻^1^, 0.016 Wh kg⁻^1^, 101.75 W kg⁻^1^) due to the lower contribution of polypyrrole in the copolymer structure. Referring to Equation 3 for calculating the specific capacitance in two-electrode systems, these results show that the sample containing a higher amount of Ppy (1:3) performs better in both basic (KOH) and semi-acidic (Na₂SO₄:H₂SO₄) media. This is due to the combination of the quasi-capacitive advantages of polyaniline (dependent on protonation) and the high ion exchange ability of polypyrrole in different media. In conclusion, although KOH may be challenging for some conductive polymers, the copolymer blend designed in this study in both types of electrolytes has shown to be a stable, adaptable, and highly efficient structure for application in supercapacitors (Fig. S4).

All values ​​of energy density (measured in Wh kg^-1^) and power density (measured in W kg^-1^) for each electrode were calculated using Equations (5) and (6) to find the best electrode materials for supercapacitor application^36^^,^^37,38^. The power density and energy density of prepared electrodes are listed in Table 1. ACP@PANI-*co*-Ppy (1:3) composite electrode exhibits a higher power density and energy density than other electrodes, indicating its superior potential for practical applications. In Equations (5) and (6), specific capacity (C_S_), working voltage window (ΔV) and discharge time (Δt) are shown.

5 $${\mathrm{E}}_{\mathrm{x}}=\frac{({\mathrm{C}}_{\mathrm{s}}\Delta {\mathrm{V}}^{2})}{2\times 3.6}$$

6 $${\mathrm{P}}_{{\mathrm{x}}} = \frac{{3600{\text{ E}}_{{\mathrm{x}}} }}{{\Delta {\mathrm{t}}}}$$

**Table 1:** Energy density value (Wh kg^-1^) as well as power density value (W kg^-1^) as well as measured for prepared electrodes.

**Electrode**	**Power density** **(W Kg**^**-1**^**)**	**Energy density** **(Wh Kg**^**-1**^**)**
**ACP**	309.91	1.2
**ACP@PANI-*co*-Ppy (1:1)**	309.92	6.5
**ACP@PANI-*co*-Ppy (1:3)**	312.2	6.7

One of the primary techniques to investigate the fundamental behavior of supercapacitors is EIS analysis. The cycle stability and rate capability of supercapacitors were directly affected by charge transfer and ion diffusion at the electrode-electrolyte interface^39^. EIS diagrams were used to investigate the electron transportation properties in ACP, ACP@PANI-*co*-Ppy (1:1), and ACP@PANI-*co*-Ppy (1:3) electrodes during the faradic reactions. Furthermore, the results are well illustrated in Fig. 7E. In the low-frequency region, the ACP@PANI-*co*-Ppy (1:3) electrode shows a steeper straight line, indicating a lower diffusion resistance compared to ACP and ACP@PANI-*co*-Ppy (1:1). The analysis of the equivalent series resistance (R_s_) of electrode with distance assessed at the junction of the curve and real axis can be seen based on the data display in Fig. 7F and the equivalent circuit parameters in the Zview software are explained in the supporting information. As the distance approaches zero, the internal resistance of the electrode decreases. The solution resistance (R_s_) remained low (around 0.2 Ω) for both electrodes, confirming the high ionic conductivity of the 3 M KOH electrolyte. However, the ACP@PANI-co-Ppy (1:3) composite exhibited a higher charge-transfer resistance (R_ct_ = 433 Ω) compared to pristine ACP. This is attributed to the initial resistance of the dense copolymer layer, which requires a few electrochemical cycles for full ionic activation and electrolyte penetration into the polymer bulk. Also, charge transfer resistance between the electrolyte solution and the electrode with a semicircle diameter at a high frequency is shown in the inner diagram of Fig. 7E. The solution resistance (R_s_) for the ACP electrode is 0.2 Ω. The charge-transfer resistance (R_ct_) of 433 Ω for the initial composite cycles is attributed to the dense polymer layer requiring ionic activation. ACP@PANI-*co*-Ppy (1:1) composite electrode showed a higher charge transfer resistance compared with the ACP and ACP@PANI-*co*-Ppy (1:3) electrodes. This shows that the electron transfer rate in the ACP@PANI-*co*-Ppy (1:1) electrode is relatively fast, which indicates better conductivity compared with our previous work^30,40^. The ACP matrix acts as a mechanical buffer, effectively accommodating the volumetric strain of the copolymer during doping/de-doping processes, which is the key technical novelty of this work^41^.

### Asymmetrical two-electrode operation

**Fig. 8:**
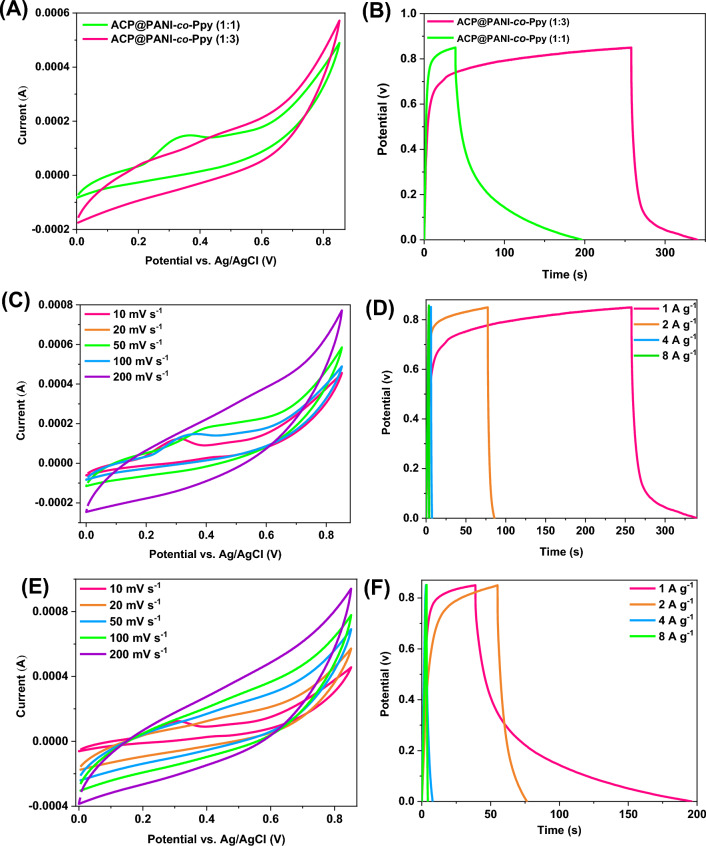

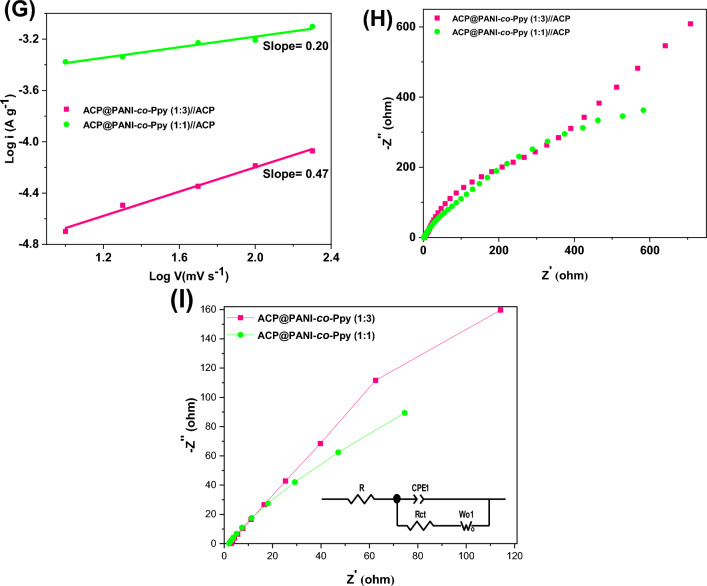
Electrochemical tests were performed in a 2-electrode system with KOH electrolyte (3.0 M). (**A**) CV curves of the prepared compounds at a scan rate of 20 mV s^-1^, (**B**) GCD curves of the compounds at 1 A g^-1^. (**C**) CV curves of ACP@PANI-*co*-Ppy(1:1)//ACP, (**D**) GCD curves of ACP@PANI-*co*-Ppy(1:1)//ACP. (**E**) CV curves of ACP@PANI-*co*-Ppy(1:3)//ACP, (**F**) GCD curves of ACP@PANI-*co*-Ppy(1:3)//ACP (**G**) Log i vs. log ν plots, (**H**) Nyquist plots of all prepared compounds at 1–100 kHz. (**I**) The value of R represents the equivalent series resistance; R_ct_ is the charge transfer resistance in the figure).

Electrochemical measurements were performed using two-electrode cells containing two active materials coupled with ACP electrodes, namely ACP@PANI-*co*-Ppy(1:1)//ACP and ACP@PANI-*co*-Ppy(1:3)//ACP, with a 3 M KOH aqueous electrolyte. The electrochemical behavior of the assembled asymmetric supercapacitors was analyzed using CV, EIS, and GCD techniques. Fig. 8(A–H) shows the results of the two-electrode cells of the fabricated asymmetric supercapacitors whose electrochemical properties were evaluated in a potential window from 0 to 1.0 V. The CV curve of the ACP@PANI-*co*-Ppy(1:3)//ACP composite is shown in Figure 8A. For better comparison, CV measurements were also performed for ACP@PANI-*co*-Ppy(1:1)//ACP. Both CV curves show two distinct redox peak pairs, indicating the redox reactions at the electrode surface and the pseudocapacitor properties during the electrochemical process. The specific capacitance was evaluated by the CV curve using Equation (2). From the analysis of the CV plots, it is estimated that the ACP@PANI-*co*-Ppy(1:1)//ACP and ACP@PANI-*co*-Ppy(1:3)//ACP samples exhibit specific capacitance values of 5.66 and 8.52 F g^-1^ at a scan rate of 20 mV s^-1^, respectively. These results clearly show the difference in the specific capacitance value. The GCD curve of the ACP@PANI-*co*-Ppy(1:3)//ACP composite is shown in Fig. 8B. For better comparison, the GCD measurement for ACP@PANI-*co*-Ppy(1:1)//ACP was also performed. The comparison of the two electrodes at a current density of 1 A g^-1^ is presented in Fig. 8B. The ACP@PANI-*co*-Ppy (1:3) electrode exhibits the longest discharge time as well as the highest specific capacitance of 33.164 F g^-1^, which is higher than the value of 19.23 F g^-1^ for ACP@ PANI-*co*-Ppy (1:1). The CV curves in Fig. 9(C, E) for the ACP@PANI-*co*-Ppy(1:1)//ACP and ACP@PANI-*co*-Ppy(1:3)//ACP samples, respectively, maintain an almost rectangular shape without significant redox peaks with increasing voltage scan rates from 10 to 20, 50, 100, and 200 mV s^-1^. This shows that both electrodes contributed to a stable charge-discharge process over the entire scan rate range, indicating good charge storage capabilities. The charge-discharge performance of ACP@PANI-*co*-Ppy(1:1)//ACP and ACP@PANI-*co*-Ppy(1:3)//ACP samples was also investigated in Fig. 9(D, F). The ACP@PANI-*co*-Ppy(1:3)//ACP device exhibits values of 24.37, 2.45, 0.35, and 0.09 F g^-1^ at current densities of 1, 2, 4, and 8 A g^-1^, respectively, which shows better energy storage performance compared to the ACP@PANI-*co*-Ppy(1:1)//ACP sample. The ACP@PANI-*co*-Ppy(1:3)//ACP configuration proved that the combination of ACP with PANI-*co*-Ppy(1:3)s enhanced the electrochemical energy storage performance of the system. Overall, the ACP@PANI-*co*-Ppy(1:3) composite improved the ion accessibility, while the EDLC and non-Faradic redox reactions facilitated the stable charge storage. As confirmed by the GCD curves at different current densities, the quasi-capacitive characteristic was observed^42^ (Fig. 9D, F). Surface-controlled and diffusion-controlled charge storage processes can be identified by the power law relationship of Equation 7:

7 $$i=a{v}^{b}$$

Where i represents the current density, ν represents the scan rate, a and b are adjustable variables. In general, the slope of the plot of log(i) versus log(ν) at a fixed potential determines the value of b. If b≈1, the charge storage capacitance mechanism is considered to be a surface-controlled capacitive process, while b≈0.5 indicates a diffusion-controlled operation^43^. As shown in Fig. 9G, the b values for ACP@PANI-*co*-Ppy(1:1)//ACP and ACP@PANI-*co*-Ppy(1:3)//ACP are about 0.20 and 0.47, respectively, corresponding to the oxidation reaction peaks, indicating that the ACP@PANI-*co*-Ppy(1:3)//ACP sample has a more likely ion-dominated diffusion-controlled charge storage mechanism. The improvement of the b-value to 0.47 in the 1:3 sample signifies a transition toward a diffusion-controlled, "battery-like" charge storage mechanism^44^.

The two-electrode asymmetric supercapacitors ACP@PANI-*co*-Ppy(1:1)//ACP and ACP@PANI-*co*-Ppy(1:3)//ACP were tested using EIS and the resulting Nyquist plot is shown in (Fig. 9(H)). The analysis of the equivalent series resistance (R_s_) of the electrode with the evaluated distance at the junction of the curve and the real axis can be seen based on the data display in Fig. 9I, R_s_ for the ACP@PANI-*co*-Ppy(1:1)//ACP sample was measured to be 2.93 Ω and for the ACP@PANI-*co*-Ppy(1:3)//ACP sample was measured to be 1.98 Ω by Zview software. In the high frequency region, the plot includes R_s_, R_ct_, and CPE components that appear along the real axis (Z_re_ in Ω). The low frequency region shows a 45° slope that intersects the imaginary axis (Z_im_ in Ω), which is characteristic of the diffusion-controlled behavior. Also, the charge transfer resistance between the electrolyte solution and the semicircular diameter electrode at high frequency is shown in the inset of the figure^42,45^. The ACP@PANI-*co*-Ppy (1:1) composite electrode showed higher charge transfer resistance compared to the ACP and ACP@PANI-*co*-Ppy (1:3) electrodes, as was also observed in the 3-electrode sample. The ACP@PANI-*co*-Ppy(1:3)//ACP sample increased the conductivity, making this device configuration much more suitable for supercapacitor applications Table 2.

**Table 2:** Comparison of our electrode with various manufactured electrodes utilized in reported supercapacitors.

**Electrode**	**Energy density** **(Wh kg**^**-1**^**)**	**Power density** **(W kg**^**-1**^**)**	**Electrolyte**	**Cycling stability**	**Ref.**
**PP/800/PANI**	51.2	380	H_2_SO_4_, 1 M	90%, 10000 cycle	^32^
**ATSAC-6**	51.83	375	KOH, 6 M	97%, 6000 cycle	^19^
**PANI-AC**	85.06	1049	H_2_SO_4_, 1 M	87%, 1000 cycle	^9^
**AC-900**	~72	868	H_2_SO_4_, 1 M	100%, 6000 cycle	^10^
**ASNPC-1**	14.4	700	KOH, 6 M	61.9%, 10000 cycle	^46^
**OCNF**_**s-x**_	6.8	125	KOH, 6 M	100%, 10000 cycle	^47^
**NS-OPC**	53.9	6063	ZnSO_4_, 1 M	86.2%, 10000 cycle	^48^
**LKNS**	108.8	2880	ZnSO_4_, 2 M	99.72%, 20000 cycle	^49^
**ACP@PANI-*co*-Ppy (1:3)**	6.7	312.2	KOH, 3 M	99%, 2000 cycle	This work

In order to determine the structural integrity of the electrode material after electrochemical testing, X-ray diffraction (XRD) analysis was performed on the ACP@PANI-*co*-PPy (1:3) composite electrode after cycling in a mixed electrolyte of Na₂SO₄:H₂SO₄ (1 M, 1:1 volume ratio (v/v)) (Fig. S5b). The absence of any significant peak shift, intensity reduction, or new phase formation demonstrates the excellent structural stability of the hybrid material under electrochemical conditions. These findings reinforce the suitability of this material for long-life supercapacitor applications under harsh mixed-acid electrolyte environments. FE-SEM image of the ACP@PANI-*co*-PPy (1:3) composite electrode after electrochemical tests in a two-electrode system with Na₂SO₄/H₂SO₄ (1 M, 1:1 volume ratio (v/v)) electrolyte shows the favorable preservation of the porous and continuous surface structure (Fig. S6). This structural stability after testing confirms the electrode’s high efficiency and durability in energy storage applications.

## Conclusion

In summary, using bio-waste as a precursor of derived activated carbon is promising and is used to achieve a high-performance supercapacitor electrode material with sustainable development. Further improvement in the properties of carbon materials was achieved by forming composites with poly(aniline-*co*-pyrrole) through an in situ chemic oxidative polymerization technique. XRD, SEM, BET, FT-IR, TEM, as well as EDS confirmed the formation of the composite. When the ACP@PANI-*co*-Ppy (1:3) electrode was examined in a three-electrode configuration and 3 M KOH electrolyte. According to the analysis of the graphs, the value of the current density was measured to be 0.6 Ag^-1^, and the composite ACP@PANI-*co*-Ppy (1:3) has a significant specific capacitance of 48.33 F g^-1^ and also cyclic durability. It shows excellent. ACP@PANI-co-Ppy (1:3) composite preserves 99% of its primary capacity after 2000 cycles. In addition, supercapacitor electrode has a high energy density of 6.7 Wh kg^-1^ also suitable power density of 312 W kg^-1^. Also, according to EIS data, the ACP@PANI-*co*-Ppy (1:3) composite has the lowest R_ct_. This composite takes advantage of synergistic effect of ACP, PANI, and Ppy, which contributes to efficient quasi-capacitive behavior, a high conductivity, and structural stability. As a result, the composite shows suitable values ​​of reversibility, specific capacitance, power density, rate capability, and energy density.

## Supplementary Information

Supplementary Information.

## Data Availability

The datasets used or analyzed during the current study are available from the corresponding author on reasonable request.

## Declarations

### Declaration of AI-assisted technologies in the writing process

While preparing this work, the authors used AI tools (QuillBot) to improve the clarity and flow of writing. After utilizing these tools, the authors carefully revised, and edited the content to confirm accuracy and originality, taking full responsibility for the final manuscript.
