# Supplementary material for: Nature-inspired composite consisting of activated carbon derived from date palm kernel and poly(aniline-co-pyrrole) copolymer as electrode for energy storage devices
Source: Sci Rep. 2026 Apr 27;16:19288. doi: 10.1038/s41598-026-49903-3 (PMC13284376; doi:10.1038/s41598-026-49903-3)
Supplement: Supplementary file 1 — Supplementary Information. [file 41598_2026_49903_MOESM1_ESM.docx]

**Supporting Information**

**Nature-inspired composite consisting of activated carbon derived from palm kernel and poly(aniline-*co*-pyrrole) copolymer as electrode for energy storage devices**

Shayan Kalavani, Atefeh Kiani, Alireza Alizadeh, Ebrahim Ahmadi^*^

^a^Department of Chemistry, Faculty of Science, University of Zanjan, P.O. Box 45195-313, Zanjan, Iran

^b^Department of Chemistry, Institute for Advanced Studies in Basic Sciences (IASBS), Zanjan 45137-66731, Iran

The corresponding author's email address: [ahmadi@znu.ac.ir](mailto:ahmadi@znu.ac.ir)

Tel: (+98) 24 3305-2269

Fax: (+98) 24 3305-2477

Pyrrole oxidation [1]:

Aniline oxidation [2]:

Random copolymerization of aniline and pyrrole:

**Figure S1.** Copolymerization of aniline and pyrrole.

**FT-IR spectrum of aniline and pyrrole copolymer**

In polyaniline, the peak at 1579 cm^−1^ is related to the quinone C=C and benzenoid stretching vibrations. The aromatic amine stretching deformation peaks at 1293 and 1099 cm^−1^ are related to the C−N and C=N (−N=quinoid=N−) stretching, respectively. A broad peak at 3432 cm^−1^ is related to the N−H stretching vibration and a peak at 823 cm^−1^ is related to the aromatic ring C−H vibration. Polypyrrole shows a sharp peak of the N−H stretching vibration band at 3431 cm^−1^. A clear peak of the symmetric and asymmetric C−H stretching vibrations of the saturated hydrocarbons of Ppy was shown at 2856 cm^−1^ compared to a small peak of the aliphatic C−H bonds observed at 2923 cm^−1^. The stretching vibration for C-C bonds is observed at 1549 cm^−1^, while a sharp peak of C-N bonds stretching vibration of pyrrole was observed at 1308 and 1182 cm^−1^. The C-H band appeared at 1037 cm^−1^. In PANI@Ppy, the spectra show the characteristic band of quinine nitrogen at 1579 cm^−1^. Bands appear at 1293 cm^−1^ and 1099 cm^−1^, which are related to the benzene ring and C−N and C=N stretching vibrations. In addition, the band at 3432 cm^−1^ is related to the C-H stretching vibration. The peak at 788 cm^−1^ belongs to the C−H of PANI ring [3-5].

**Figure S2**. FT-IR spectra of PANI, Ppy, and their copolymer.

**Redox reactions for polyaniline and polypyrrole**

Polyaniline (PANI) and polypyrrole (Ppy) are regarded as important conducting polymers due to their ability to undergo reversible redox reactions in aqueous environments. Polyaniline (PANI) undergoes oxidation and reduction transitions when incorporated into 3 M KOH, shifting between its oxidation states: leucoemeraldine (reduced), emeraldine (semi-oxidized) and pernigraniline (fully oxidized). This transition between oxidation states leads to the high quasi-capacitive capacitance of polyaniline, while its porous structure facilitates the efficient exchange of K⁺ and OH⁻ ions. On the other hand, polypyrrole also has a remarkable capacity to absorb and release electrolyte ions due to its conjugated structure and high electronic stability. In KOH solution, K⁺ and OH⁻ ions play a key role in the charge balance during the doping/dedoping process. In the present experiment, in addition to the alkaline electrolyte, a new mixed electrolyte system consisting of Na₂SO₄:H₂SO₄ (1 M, 1:1 volume ratio (v/v)) was also used. This semi-acidic environment with Na⁺, H⁺ and SO₄²⁻ ions show a different behavior in the interaction with the copolymer chain. In this system, protons (H⁺) with high reactivity play an important role in the oxidation process of polyaniline, and SO₄²⁻ can also enter the chain as a charge-compensating anion during the doping process. At the same time, Na⁺ ions are helpful during redox reactions by moving inside Ppy and raising the capacity for storing charges. In this copolymer, all the PANI and Ppy segments play together during the oxidation and reduction which boosts energy storage, improves how ions move and reduces resistance in the polymer. Having PANI-*co*-Ppy copolymer in the electrolytes (whether alkaline or semi-acidic) helps the electrode improve its performance thanks to its coordinated and proper redox activity.

1. Polyaniline [6]:

1. Polypyrrole [7]:

**Figure S3.** Redox reactions for polyaniline and polypyrrole

**Electrochemical characterization**

|   **(a)** |   **(d)** |
| --- | --- |
|   **(b)** |   **(e)** |
|   **(c)** |   **(f)** |

**Figure S4.** (a-c) Electrochemical tests (CV, GCD, EIS) in two electrode system with Na₂SO₄:H₂SO₄ (1 M, 1:1 volume ratio (v/v)) electrolyte, (d-f) Electrochemical tests (CV, GCD, EIS) in three electrode system with KOH (3 M) electrolyte.

**Equivalent circuit parameters in Zview software**

Zview software was used to fit the EIS diagram and draw the equivalent circuit of the ACP@PANI-co-Ppy(1:3) electrode. The EIS diagram was fitted with a Chi-Squared value of 0.0079402. The R_s_ value is 0.2 Ω, and the R_ct_ value is 1.98 Ω for the optimized composite. The low R_s_ and R_ct_ values confirm the high ionic conductivity of the 3 M KOH electrolyte and the efficient charge-transfer kinetics at the electrode/electrolyte interface. The C_dl_ value is 628.52 F, and the CPE value is divided into two parts: CPE-T (0.0010661) and CPE-P (0.78647). The Warburg impedance (W) parameters are W-R (870.3), W-T (0.0059795), and W-P (15.16 Ω s^-0.5^). The higher C_dl_ value and lower R_ct_ contribute to the enhanced capacity and fast power delivery. In Fig. 7F, the absence of a large semicircle confirms that the supercapacitor exhibits minimal resistance to Faradaic redox reactions, facilitating rapid energy storage.

**XRD test after electrochemical tests**

In order to determine the structural integrity of the electrode material after electrochemical testing, X-ray diffraction (XRD) analysis was performed on the ACP@PANI-*co*-PPy (1:3) composite electrode after cycling in a mixed electrolyte of Na₂SO₄:H₂SO₄ (1 M, 1:1 volume ratio (v/v)) (Fig. S5b). It is typical for a semi-crystalline polyaniline (PANI) and polypyrrole (PPy) to have a broad peak in the (2θ) ~20° region. The presence of this peak after 1000 charge-discharge cycles indicates that the backbone of the conducting polymers remains intact, despite the redox activity and ion intercalation/deintercalation stress during cycling. Additionally, a weak and broad diffraction region between 25° and 30° is observable, which is assigned to the amorphous carbon structure of activated carbon powder (ACP). The absence of any significant peak shift, intensity reduction, or new phase formation demonstrates the excellent structural stability of the hybrid material under electrochemical conditions. The combination of XRD results and electrochemical cycling stability (>98% capacitance retention after 1000 cycles) strongly supports the mechanical and chemical resilience of the ACP@PANI-*co*-PPy (1:3) composite. These findings reinforce the suitability of this material for long-life supercapacitor applications under harsh mixed-acid electrolyte environments. We trust this additional data addresses the reviewer’s concern regarding the structural integrity of the electrode post-cycling.

|   **(a)** |   **(b)** |
| --- | --- |

**Figure S5**. XRD pattern of ACP@PANI-*co*-PPy (1:3) composite electrode (a) before and (b) after electrochemical tests.

**FE-SEM images before and after electrochemical testing**

FE-SEM image of the ACP@PANI-*co*-PPy (1:3) composite electrode after electrochemical tests in a two-electrode system with Na₂SO₄/H₂SO₄ (1 M, 1:1 volume ratio (v/v)) electrolyte shows the favorable preservation of the porous and continuous surface structure. Despite the electrode being exposed to electrochemical stress conditions resulting from the charging and discharging cycles, the overall structure of the ZnCl₂ activated carbon matrix, along with the PANI-*co*-PPy copolymer, remained largely stable. Microstructural observations indicate the presence of a porous and relatively uniform network, which is very favorable for the effective penetration of electrolyte ions. Only slight local damage to the surface, including limited micron cracks and minor compactions in some areas, was observed, which could be related to the expansion-contraction phenomena caused by the doping and deadening of the polymers during the tests. This structural stability after testing confirms the electrode's high efficiency and durability in energy storage applications.

|   **(a)** | 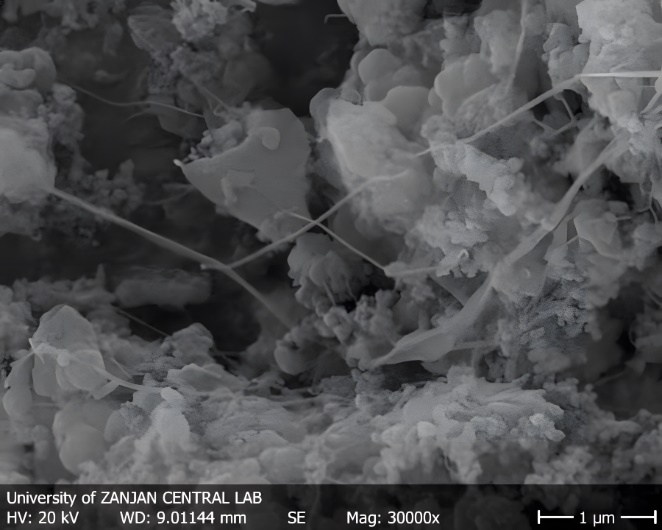  **(b)** |
| --- | --- |

**Figure S6.** FE-SEM images of the ACP@PANI-*co*-PPy (1:3) composite electrode (a) before and (b) after electrochemical tests, two-electrode electrochemical testing in Na₂SO₄/H₂SO₄ (1 M, 1:1 volume ratio (v/v)) electrolyte.

**Specific capacitance from galvanostatic charge-discharge (GCD) data**

Below, a detailed step-by-step derivation of the equation used in the paper is presented.

The total capacitance is defined by the basic relation between charge Q and voltage V [8]:

$$C= \frac{Q}{\Delta V}$$

In a galvanostatic process (constant current), the charge Q delivered during the discharge process is given by:

$$Q= \Delta t . I$$

Where ( I ) is the discharge current (A), and ( ∆t ) is the discharge time (s).

Combining the Two Equations:

Substitute Q into the capacitance definition:

$$C= \frac{I . \Delta t}{\Delta v}$$

Specific Capacitance (per unit mass):

To obtain specific capacitance Csp (in F g^-1^), we divide by the mass of the active material m:

$$C= \frac{I . \Delta t}{m . \Delta V}$$

This is the expression used in our manuscript as Equation (3), and it is valid under constant current conditions, which apply in our GCD measurements.

in systems where significant pseudocapacitive or Faradaic behavior causes the GCD curve to become non-linear, the voltage profile does not follow a perfect triangular shape. In such cases, an integral-based approach for total charge calculation:

$$Q= \int I \left( t \right)\mathrm{dt}$$

in constant current discharge, I(t)=I_0_​, the expression simplifies to:

$$Q=I_{0}. \Delta t$$

Therefore, the simplified equation used holds even in quasi-capacitive systems, as long as the current is constant [9].

For the two-electrode system, the specific capacitance of the material (C_s_) is derived as C_s_ = 4 × C_device_ according to standard electrochemical connections for symmetric cells [10].

$$C_{device}=\frac{(I \times\Delta t)}{(M \times\Delta V)}$$

**Table S1:** Comparison of electrochemical performance and cycling stability of neat conducting polymers vs. carbon-based composites in alkaline/acidic electrolytes.

| Electrode material | Electrolyte | Specific capacitance (F g^-1^) | Stability (retention %) | Cycle number | Ref. |
| --- | --- | --- | --- | --- | --- |
| Neat PANI | 1 M KOH | 15.45 | < 35% | 500 | [7] |
| Neat Ppy | 3 M KOH | 931.0* | < 60% | 1000 | [11] |
| PANI/MWCNTs | 1M H_2_SO_4_ | 320.0 | 8% | 50 | [12] |
| PANI-co-PPy | 1M H_2_SO_4_ | 227.0 | 65% | 1000 | [13] |
| AC/PANI | 6 M KOH | 180.0 | 91% | 1000 | [14] |
| RCS-AC (Biomass) | 3 M KOH | 137.0 | 97.2% | 5000 | [15] |
| ACP@PANI-co-Ppy (1:3) | 3 M KOH | 65.03 | 99.5% | 2000 |  |

*Measured at a very low scan rate (5Mv s^-1^).

As summarized in Table S1, neat conducting polymers (PANI and PPy) exhibit significantly lower performance and poor structural stability in alkaline media (KOH) compared to acidic environments. PANI, in particular, undergoes de-protonation at pH > 9, transitioning from the conductive emeraldine salt to the emeraldine base form, which severely limits its charge-transfer kinetics. Furthermore, without a rigid support, these polymers suffer from over 40% capacity loss due to mechanical pulverization and structural collapse during ion doping/de-doping cycles.

In contrast, our ACP@PANI-co-Ppy (1:3) composite utilizes the date palm kernel-derived carbon as a "Mechanical Buffer". The ACP matrix successfully accommodates the volumetric strain of the copolymer chains within its rigid hierarchical pores. This structural synergy, combined with the higher PPy content which maintains electronic stability in alkaline conditions, results in a near-perfect retention of 99.5% in 3 M KOH. This proves that the proposed hybrid architecture is essential for achieving industrial-grade durability in supercapacitor applications.

**References**

1. Zare, E.N., et al., *Electroconductive multi-functional polypyrrole composites for biomedical applications.* Applied Materials Today, 2021. **24**: p. 101117.

2. Sapurina, I. and J. Stejskal, *The mechanism of the oxidative polymerization of aniline and the formation of supramolecular polyaniline structures.* Polymer international, 2008. **57**(12): p. 1295–1325.

3. Al-Obaidi, N., Z. Al-Garawi, and A. Al-Mahdawi. *Polyaniline doping with nanoparticles: A review on the potential of electrical properties*. in *Journal of Physics: Conference Series*. 2021. IOP Publishing.

4. Diauudin, F.N., et al., *Preparation and Characterisation of Polypyrrole‐Iron Oxyhydroxide Nanocomposite as Sensing Material.* Advances in Materials Science and Engineering, 2020. **2020**(1): p. 8762969.

5. Bekhoukh, A., et al., *New hybrid adsorbents based on polyaniline and polypyrrole with silicon dioxide: synthesis, characterization, kinetics, equilibrium, and thermodynamic studies for the removal of 2, 4-dichlorophenol.* Polymers, 2023. **15**(9): p. 2032.

6. Song, Y., *The adsorption of amoxicillin by mesoporous silica supported polymers*. 2018, Rutgers University-School of Graduate Studies.

7. Maksymiuk, K., *Chemical reactivity of polypyrrole and its relevance to polypyrrole based electrochemical sensors.* Electroanalysis: An International Journal Devoted to Fundamental and Practical Aspects of Electroanalysis, 2006. **18**(16): p. 1537–1551.

8. Sharma, S. and P. Chand, *Supercapacitor and electrochemical techniques: A brief review.* Results in Chemistry, 2023. **5**: p. 100885.

9. Conway, B.E., *Electrochemical supercapacitors: scientific fundamentals and technological applications*. 2013: Springer Science & Business Media.

10. Sundriyal, S., et al., *Metal-organic frameworks and their composites as efficient electrodes for supercapacitor applications.* Coordination Chemistry Reviews, 2018. **369**: p. 15–38.

11. Khan, S., et al., *Electrochemical investigation of PANI: PPy/AC and PANI: PEDOT/AC composites as electrode materials in supercapacitors.* Polymers, 2022. **14**(10): p. 1976.

12. Liu, T., et al., *Polyaniline and polypyrrole pseudocapacitor electrodes with excellent cycling stability.* Nano letters, 2014. **14**(5): p. 2522–2527.

13. Chonat, A. and S. Palatty, *Enhanced electrochemical performance of a hybrid supercapacitive material based on ternary doped polyaniline/activated carbon composite.* Energy & Fuels, 2020. **34**(8): p. 10148–10159.

14. Dubey, P., et al., *Facile synthesis of pineapple waste-derived carbon and polyaniline composite for high-energy-density asymmetric supercapacitors.* Energy & Fuels, 2023. **37**(12): p. 8659–8671.

15. Rajasekaran, S.J., et al., *Investigation of different aqueous electrolytes for biomass-derived activated carbon-based supercapacitors.* Catalysts, 2023. **13**(2): p. 286.
